# A clinical model to predict distant metastasis in patients with superficial gastric cancer with negative lymph node metastasis and a survival analysis for patients with metastasis

**DOI:** 10.1002/cam4.3680

**Published:** 2020-12-22

**Authors:** Jingyu Chen, Lunpo Wu, Zizhen Zhang, Sheng Zheng, Yifeng Lin, Ning Ding, Jiawei Sun, Liuhong Shi, Meng Xue

**Affiliations:** ^1^ Department of Gastroenterology The Second Affiliated Hospital of Zhejiang University School of Medicine Hangzhou China; ^2^ Institution of Gastroenterology Zhejiang University Hangzhou China; ^3^ Department of Ultrasound The Second Affiliated Hospital of Zhejiang University School of Medicine Hangzhou China

**Keywords:** distant metastasis, superficial gastric cancer, nomogram, SEER Program

## Abstract

**Background:**

Distant metastasis (DM) is relatively rare in superficial gastric cancer (SGC), especially in patients without lymph node metastasis. This study aimed to explore the main clinical risk factors for DM in patients with superficial gastric cancer‐no lymph node metastasis (SGC‐NLNM) and the prognostic factors for patients with DM.

**Methods:**

Records of patients with SGC‐NLNM between 2004 and 2015 were collected from the public Surveillance, Epidemiology, and End Results (SEER) database. Both univariate and multivariate logistic regressions were performed to analyze the clinical risk factors for DM. The Kaplan–Meier method and Cox regression model were used to identify prognostic factors for patients with DM. A nomogram was built based on multivariate logistic regression and evaluated by the C‐index, the calibration, and the area under the receiver operating characteristic curve (AUC).

**Results:**

We developed and validated a nomogram to predict DM in patients with SGC‐NLNM, showing that race, age, primary site, depth, size, and grade were independent risk factors. The built nomogram had a good discriminatory performance, with a C‐index of 0.836 (95% confidence interval [CI]: 0.813–0.859). Calibration plots showed that the predicted DM probability was identical to the actual observations in both the training and validation sets. AUC was 0.846 (95% CI: 0.820–0.871) and 0.801 (95% CI: 0.751–0.850) in the training and validation sets, respectively. The results of the survival analysis revealed that surgery (hazard ratio [HR] = 0.249; 95% CI, 0.125–0.495), chemotherapy (HR = 0.473; 95% CI, 0.353–0.633), and grade (HR = 1.374; 95% CI, 1.018–1.854) were independent prognostic factors associated with cancer‐specific survival (CSS), but radiotherapy was not (log‐rank test, *p* = 0.676).

**Conclusions:**

We constructed a sensitive and discriminative nomogram to identify high‐risk patients with SGC‐NLNM who may harbor dissemination at initial diagnosis. The tumor size and primary site were the largest contributors to DM prediction. Compared with radiotherapy, aggressive surgery, and chemotherapy may be better options for patients with DM.

## Graphical abstract

We identified the main clinical risk factors for distant metastasis (DM) in superficial gastric cancer‐no lymph node metastasis patients and the prognostic factors for the metastatic patients. Besides, a clinical tool was built for the DM prediction.

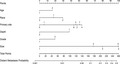

## 1 INTRODUCTION

Gastric cancer (GC) is a common gastrointestinal malignant tumor, causing a large number of deaths every year and imposing a huge burden on both family and society.^1^, ^2^ Although the incidence of GC has declined over the past few decades, GC remains the third most common cause of cancer‐related deaths worldwide.^3^ Thanks to the universal screening and improved endoscopy techniques in recent years, the diagnosis rate of early gastric cancer (EGC) has increased rapidly. EGC is defined as a superficial gastric lesion confined to the mucosa (T1a) and submucosa (T1b), regardless of the lymph node status.^4^ Patients with superficial gastric cancer‐no lymph node metastasis (SGC‐NLNM), who have less than 1% possibility of lymph node metastasis (LNM), are considered as absolutely appropriate candidates for endoscopic therapy (ET), such as endoscopic mucosal resection (EMR) and endoscopic submucosal dissection (ESD).^5^ Encouragingly, the 5‐year overall survival rate and cancer‐specific survival rates of patients with EGC can reach over 90%.^6^

Distant metastasis (DM) is a crucial point in the management of malignant tumors. As the main characteristics of advanced GC, DM is always associated with poor survival.^7^ Due to the lack of specific manifestations at an early stage, a large proportion of patients with GC have DM when diagnosed. Several studies revealed that about 40% of patients with newly diagnosed GC had synchronous DM at initial diagnosis.^8^, ^9^ Recently, based on the clinicopathological characteristics, a nomogram was constructed to predict peritoneal dissemination in patients with GC, and the area under the receiver operating characteristic curve (AUC) was 0.791 (95% CI: 0.762–0.820).^10^ Although the probability of DM in patients with SGC‐NLNM is relatively low, there are still cases of SGC that skipped LNM and directly metastasized to distant organs. Intriguingly, a recent study showed that patients with SGC‐NLNM who have DM had a worse prognosis when compared with patients with T1N+M1 GC because of more aggressive behaviors.^11^ However, the main risk factors for DM in patients with SGC‐NLNM and prognostic factors for patients with DM are both poorly determined due to the limited number of cases.

In this study, we used the data extracted from the Surveillance, Epidemiology and End Results (SEER) database to identify these factors. In addition, a nomogram was constructed to predict DM in patients with SGC‐NLNM, which would be useful in screening metastatic patients and guiding optimal treatment.

## 2 METHODS

### 2.1 Patients

The data of patients with T1N0Mx GC from the SEER database were collected using the SEER*Stat software (version 8.3.6; www.seer.cancer.gov) with a private ID (account number: 11629‐Nov2019), and treatment data were obtained from SEER custom data via further application. Informed consent was not required because the SEER database is publicly available.^12^

The inclusion criteria were as follows: (1) patients with T1N0Mx GC aged over 18 years who were diagnosed between 2004 and 2015; (2) patients who were diagnosed with positive histology and GC as the only type of primary cancer; (3) patients who had GC with a clear depth of invasion confined to the mucosa (T1a) or submucosa (T1b) and were free of LNM; (4) patients with complete records of cancer‐specific survival (CSS) and survival months.

### 2.2 Variables

The following variables were selected from the SEER database: patient ID, sex, age at diagnosis, year of diagnosis, marital status, primary site, race, histology, grade, TNM stage (the 6th American Joint Committee on Cancer stage system), cancer stage (including tumor size and tumor extension), surgery, radiation, chemotherapy, survival months, and CSS.

Race was classified into white, black, or other; sex was recorded as male or female; age was regrouped into ≤60 years old and >60 years old; year of diagnosis was divided into 2004–2009 and 2010–2015; primary site of the tumor was grouped into seven different parts: gastric body, antrum/pylorus, lesser curve, greater curve, cardia, fundus, and overlapping/not otherwise specified (NOS). Grade was grouped into well/moderately differentiated and poorly differentiated/undifferentiated. As for the tumor size, all cases were divided into five groups: ≤2 cm, ≤3 cm (2 cm < tumor size ≤3 cm), ≤5 cm (3 cm < tumor size ≤5 cm), >5 cm, and diffuse/unknown (cannot be assessed). Invasion depth was grouped as mucosa (T1a) and submucosa (T1b). Histology information was classified into intestinal type or diffuse type according to Lauren type: patients with GC who had histologically confirmed signet ring cell carcinoma (code 8490), carcinoma‐diffuse type (code 8145), and linitis plastica (code 8142) were classified as diffuse type, while tubular adenocarcinoma (code 8211), adenocarcinoma‐intestinal type (code 8144), papillary adenocarcinoma (code 8260), and adenocarcinoma‐NOS (code 8140) were classified as intestinal type. Surgery was grouped as “yes” or “no”; chemotherapy and radiation was grouped as “yes” or “no/unknown” according to the SEER program. CSS was defined as the time from diagnosis to the date of death due to GC.

### 2.3 Statistical analysis

In this study, univariate and multivariate logistic regression analyses were carried out to analyze the risk factors for DM in patients with SGC‐NLNM (presenting as odds ratio [OR] with 95% confidence intervals [CI]). A nomogram was constructed based on the results of multivariate logistic regression, and its performance was assessed by the C‐index, calibration, and AUC. Calibration was presented graphically by plotting the association between the predicted probability and the actual outcome. The model fit was assessed using the Hosmer–Lemeshow test. To validate the nomogram, all included patients with SGC‐NLNM were randomly grouped into the training set (70%) and validation set (30%). During the external validation, the total score of each SGC‐NLNM patient in the validation set was calculated according to the established nomogram. For survival analysis, the Kaplan–Meier method and Cox regression model were utilized to analyze the CSS in T1N0M1 patients. All statistical analyses were performed using STATA 16.0 software and R software (http://www.r‐project.org, version 3.6.0). Difference were considered statistically significant for a two‐sided *p* < 0.05.

## 3 RESULTS

### 3.1 Patient baseline characteristics

A total of 3293 patients with SGC‐NLNM were included in our study. Among them, 269 (8.17%) had DM, and the remaining 3024 (91.83%) had no DM. From the perspective of longitudinal data, patients with DM were older, more often male, and more often white. In addition, the lesion often located in the cardia, gastric body, and overlapping/NOS; presented in T1a patients; had a worse differentiated grade; and had a larger tumor size. The detailed baseline characteristics of the patients with DM are presented in Table 1.

**TABLE 1:** Baseline clinical characteristics of SGC‐NLNM patients in our study

Variable	M0		M1	
	(*n* = 3024)	%	(*n* = 269)	%
Age at diagnosis				
≤60	856	28.31	106	39.41
>60	2168	71.69	163	60.59
Gender				
Female	1139	37.67	94	34.94
Male	1885	62.33	175	65.06
Race				
White	1909	63.13	188	69.89
Black	362	11.97	49	18.22
Other	753	24.90	32	11.90
Marriage				
Married	1844	60.98	162	60.22
Unmarried	1040	34.39	100	37.17
Unknown	140	4.63	7	2.60
Primary site				
Antrumy/Pylorus	911	30.13	32	11.90
Body	312	10.32	36	13.38
Cardia	972	32.14	106	39.41
Fundus	85	2.81	16	5.95
Lesser curvature	304	10.05	10	3.72
Greater curvature	112	3.70	10	3.72
Overlapping/Nos	328	10.85	59	21.93
Histology				
Intestinal type	2379	78.67	207	76.95
Diffuse type	645	21.33	62	23.05
Grade				
Well/Moderate	1651	54.60	105	39.03
Poorly/Undifferentiated	1373	45.40	164	60.97
Tumor size				
≤2 cm	1543	51.03	22	8.18
≤3 cm	439	14.52	32	11.90
≤5 cm	321	10.62	33	12.27
>5 cm	135	4.46	26	9.67
Diffuse/Unknown	586	19.38	156	57.99
Depth				
T1a	1569	51.88	210	78.07
T1b	1455	48.12	59	21.93
Year of diagnosis				
2004–2009	1381	45.67	97	36.06
2010–2015	1643	54.33	172	63.94

Note

≤3 cm, 2 cm < tumor size ≤ 3 cm; ≤5 cm, 3 cm < tumor size ≤ 5 cm.

Abbreviations: Nos, not otherwise specified; SGC‐NLNM, superficial gastric cancer‐no lymph node metastasis.

### 3.2 Risk analysis of DM in patients with SGC‐NLNM

**FIGURE 1:**
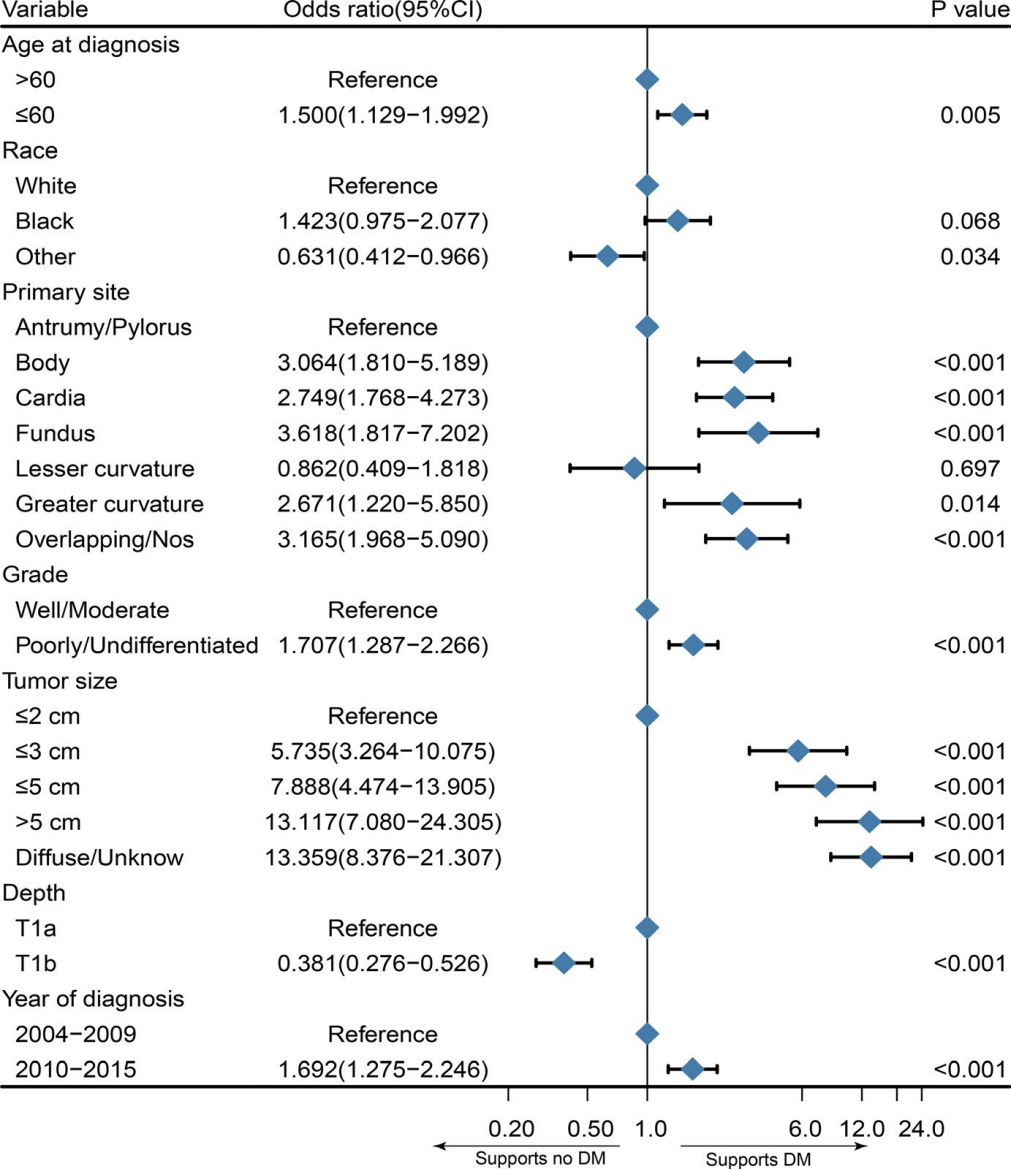
Forest plot for the potential risk factors for distant metastasis in patients with superficial gastric cancer‐no lymph node metastasis

To further explore the risk factors of DM in patients with SGC‐NLNM, we performed univariate logistic regression to identify significant candidate factors for DM and multivariate logistic regression to adjust for confounding factors. In the univariate model, a larger tumor size (*p* < 0.001), poorer differentiated grade (*p* < 0.001), younger age (*p* < 0.001), year of diagnosis between 2010 and 2015 (*p* = 0.003), and depth of T1a (*p* < 0.001) were significantly associated with a higher risk of DM. In terms of the primary site, gastric body (*p* < 0.001), cardia (*p* < 0.001), fundus (*p* < 0.001), greater curve (*p* < 0.001), and overlapping/NOS (*p* < 0.001) tended to have a higher risk of DM than the antrum/pylorus, while the lesser curve (*p* = 0.707) had a risk of DM comparable to that of the antrum/pylorus. In addition, we also found that there was no significant association between Lauren type, sex, marriage, and DM. The detailed results are presented in Table 2. Then, significant variables (*p* < 0.05) from the univariate model were selected to adjust for potential confounding factors and further analyze the association between different variables and the risk of DM. The results of multivariate logistic analysis (Figure 1) further confirmed that age, year of diagnosis, race, size, primary site, grade, and depth were independent risk factors for DM in patients with SGC‐NLNM.

**TABLE 2:** Univariate and multivariate logistic regression analysis to identify risk factors for DM in SGC‐NLNM patients

Variable	Univariate analysis			Multivariate analysis		
	OR	95% CI	*p* value	OR	95% CI	*p* value
Age at diagnosis						
>60	Ref	—	—	Ref	—	—
≤60	1.647	1.274–2.130	**<0.001**	1.5	1.129–1.992	**0.005**
Gender					NI	
Female	Ref	—	—			
Male	1.125	0.866–1.461	0.377			
Race						
White	Ref	—	—	Ref	—	—
Black	1.374	0.984–1.919	0.062	1.423	0.975–2.077	0.068
Other	0.432	0.294–0.634	**<0.001**	0.631	0.412–0.966	**0.034**
Marriage					NI	
Married	Ref	—	—			
Unmarried	1.094	0.843–1.420	0.497			
Unknown	0.569	0.262–1.236	0.155			
Primary site						
Antrumy/Pylorus	Ref	—	—	Ref	—	—
Body	3.285	2.006–5.379	**<0.001**	3.064	1.810–5.189	**<0.001**
Cardia	3.105	2.070–4.657	**<0.001**	2.749	1.768–4.273	**<0.001**
Fundus	5.359	2.826–10.162	**<0.001**	3.618	1.817–7.202	**<0.001**
Lesser curvature	0.936	0.455–1.927	0.859	0.862	0.409–1.818	0.697
Greater curvature	2.542	1.217–5.310	**0.013**	2.671	1.220–5.850	**0.014**
Overlapping/Nos	5.121	3.270–8.019	**<0.001**	3.165	1.968–5.090	**<0.001**
Histology					NI	
Intestinal type	Ref	—	—			
Diffuse type	1.105	0.821–1.486	0.511			
Grade						
Well/Moderate	Ref	—	—	Ref	—	—
Poorly/Undifferentiated	1.878	1.455–2.424	**<0.001**	1.707	1.287–2.266	**<0.001**
Tumor size						
≤2 cm	Ref	—	—	Ref	—	—
≤3 cm	5.112	2.941–8.888	**<0.001**	5.735	3.264–10.075	**<0.001**
≤5 cm	7.210	4.149–12.531	**<0.001**	7.888	4.474–13.905	**<0.001**
>5 cm	13.508	7.455–24.475	**<0.001**	13.117	7.080–24.305	**<0.001**
Diffuse/Unknown	18.671	11.830–29.469	**<0.001**	13.359	8.376–21.307	**<0.001**
Depth						
T1a	Ref	—	—	Ref	—	—
T1b	0.303	0.225–0.408	**<0.001**	0.381	0.276–0.526	**<0.001**
Year of diagnosis						
2004–2009	Ref	—	—	Ref	—	—
2010–2015	1.490	1.150–1.931	**0.003**	1.692	1.275–2.246	**<0.001**

Note

≤3 cm, 2 cm < tumor size ≤ 3 cm; ≤5 cm, 3 cm < tumor size ≤ 5 cm.

Abbreviations: Nos, not otherwise specified; NI, not included; OR, odd ratio; Ref: reference; SGC‐NLNM, superficial gastric cancer‐no lymph node metastasis.

Statistical significances are marked in bold.

### 3.3 Construction and validation of the nomogram to predict DM probability

**FIGURE 2:**
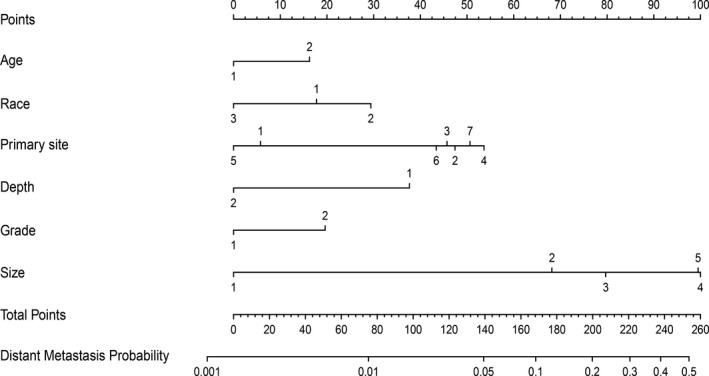
Nomogram for predicting the probability of distant metastasis. Age: 1, >60; 2, ≤60. Primary site: 1, antrum/pylorus; 2, body; 3, cardia; 4, fundus; 5, lesser curvature; 6, greater curvature; 7, overlapping/not otherwise specified. Grade: 1, well/moderate; 2, poorly/undifferentiated. Tumor size: 1, ≤2 cm; 2, ≤3 cm; 3, ≤5 cm; 4, >5 cm; 5, unknown/diffuse. Depth: 1, T1a; 2, T1b

We established a nomogram to predict the probability of DM in patients with SGC‐NLNM, incorporating age, race, primary site, tumor size, depth, and grade (Figure 2). The C‐index of the model was 0.836 (95% CI: 0.813–0.859). Beta‐coefficients from the model were used to assign scores. By summing the scores of the variables, the probability of DM was predictable for each specific patient. In our model, the tumor size and primary site were the largest contributors to DM prediction. Point and score assignment for every variable are shown in Table S1.

**FIGURE 3:**
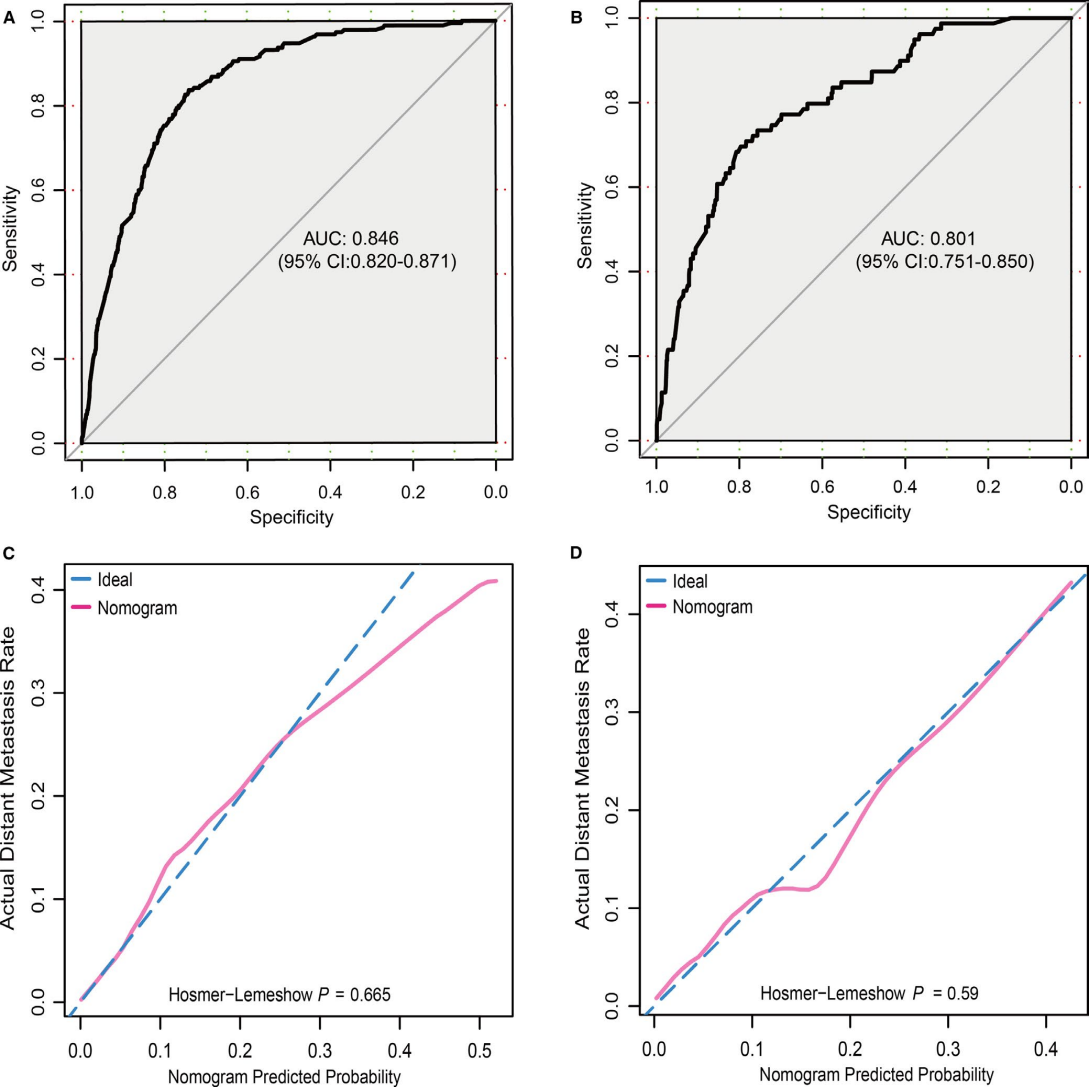
Nomogram validation. (A) Receiver operating characteristic (ROC) curve of the nomogram from the training set. The AUC is 0.846 and 95% CI 0.820–0.871. (B) ROC curve of the nomogram from the validation set. The AUC is 0.801 and 95% CI 0.751–0.850. (C) Calibration plot of the nomogram from the training set. (D) Calibration plot of the nomogram from the validation set

To test the performance of this nomogram, we performed internal and external validation. Seventy percent of patients with SGC‐NLNM were randomly grouped into the training set, while the rest were selected as the validation set. We assessed the efficacy of the proposed nomogram using the receiver operating characteristic (ROC) curves in both the training and validation sets (Figure 3A,B). The AUC for the training set was 0.846 (95% CI: 0.820–0.871). In the validation set, the total predictive score of each SGC‐NLNM patient was calculated according to the established nomogram using the training set data. The AUC for the validation set was 0.801 (95% CI: 0.751–0.850). As displayed by the calibration plots for the proposed nomogram, the predicted DM probability for the training and validation sets (Hosmer–Lemeshow test, p = 0.665, p = 0.59, respectively) (Figure 3C,D) of patients with SGC‐NLNM was identical to the actual observations. All these outcomes proved the utility of this model in predicting DM in patients with SGC‐NLNM.

### 3.4 Survival analysis of patients with metastatic SGC‐NLNM

**FIGURE 4:**
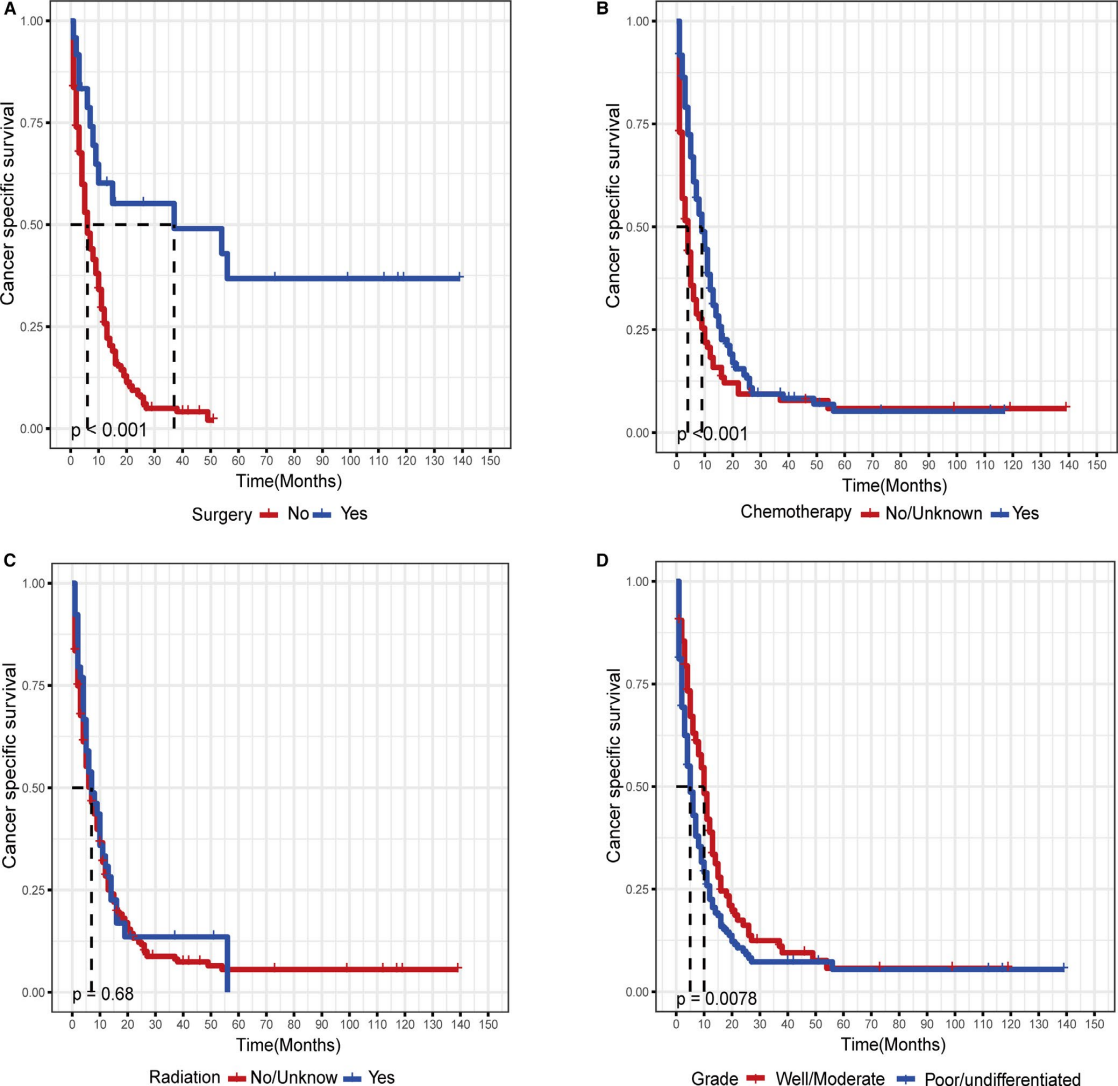
Kaplan–Meier survival curve for cancer‐specific survival in patients with metastatic superficial gastric cancer‐no lymph node metastasis, stratified by (A) surgery, (B) chemotherapy, (C) radiotherapy, and (D) grade

After exploring the risk factors of DM in patients with SGC‐NLNM, we also used the Kaplan–Meier method and Cox regression model to analyze the CSS in patients with metastatic SGC‐NLNM. Univariate analysis revealed that age, year of diagnosis, and primary tumor site (log‐rank test, *p* > 0.05) were not associated with CSS in patients with metastatic SGC‐NLNM. The addition of surgery (Figure 4A) and chemotherapy (Figure 4B) significantly improved CSS (log‐rank test, *p* < 0.001), while radiotherapy (Figure 4C) failed to improve CSS (log‐rank test, *p* = 0.676). In addition, surgery significantly improved the median CSS, while chemotherapy only improved CSS in the short term. Patients with a worse differentiated grade (Figure 4D) or a larger tumor size had shorter CSS (log‐rank test, *p* < 0.05). In addition, patients with diffuse type tumors tended to have worse CSS than those with intestinal type tumors (log‐rank test, *p* = 0.003). The detailed results of the survival analysis are shown in Table 3.

**TABLE 3:** Univariate and multivariate analysis for cancer‐specific survival (CSS) in the metastatic SGC‐NLNM patients

Variable	Univariate analysis		Multivariate analysis	
	Median survival time (months)	*p* value	HR (95% CI)	*p* value
Age at diagnosis		0.536	NI	
>60	5			
≤60	9			
Gender		0.569	NI	
Female	7			
Male	6			
Race		0.831	NI	
White	6			
Black	7			
Other	8			
Marriage		0.088	NI	
Married	6			
Unmarried	8			
Unknown	4			
Primary site		0.287	NI	
Antrumy/Pylorus	8			
Body	4			
Cardia	8			
Fundus	7			
Lesser curvature	7			
Greater curvature	5			
Overlapping/Nos	6			
Histology		**0.003**		
Intestinal type	7		Ref	
Diffuse type	5		1.324 (0.943–1.857)	0.105
Grade		**0.008**		
Well/Moderate	10		Ref	
Poorly/Undifferentiated	5		1.374 (1.018–1.854)	**0.038**
Tumor size		**0.033**		
≤2 cm	8		Ref	
≤3 ccm	10		1.218 (0.628–2.363)	0.56
≤5 cm	6		1.283 (0.676–2.437)	0.445
>5 cm	6		1.544 (0.787–3.027)	0.206
Diffuse/Unknown	6		1.552 (0.884–2.725)	0.126
Depth		**<0.001**		
T1a	6		Ref	
T1b	9		0.823(0.572–1.183)	0.292
Surgery		**<0.001**		
No	6		Ref	
Yes	37		0.249(0.125–0.495)	**<0.001**
Chemotherapy		**<0.001**		
No/unknown	4		Ref	
Yes	9		0.473(0.353–0.633)	**<0.001**
Radiation		0.676	NI	
No/unknown	7			
Yes	7			
Year of diagnosis		0.910	NI	
2004–2009	6			
2010–2015	7			

Note

≤3 cm, 2 cm < tumor size≤3 cm; ≤5 cm, 3 cm < tumor size≤5 cm.

Abbreviations: NI, not included; Nos, not otherwise specified; OR, odd ratio; Ref, reference.

Statistical significances are marked in bold.

Then, multivariate Cox regression was performed to adjust for confounding factors. All of the statistically potential independent factors (age, tumor size, histology, depth, surgery, chemotherapy) selected by univariate analysis were incorporated into the multivariate Cox model. As shown in Table 3, the results of the Cox regression model revealed that surgery (HR = 0.249; 95% CI, 0.125–0.495), chemotherapy (HR = 0.473; 95% CI, 0.353–0.633), and advanced grade (HR = 1.374; 95% CI, 1.018–1.854) were independent factors associated with CSS. However, the histology of diffuse type, tumor size, and invasion depth of T1a were no longer positive predictors (*p* > 0.05).

## 4 DISCUSSION

With the development of endoscopic technology, risk factors of LNM in SGC have been a hot area of research for many years. Because of this work, consensus on ESD and EMR for EGC has been well established; however, DM in patients with SGC‐NLNM has rarely been described. To the best of our knowledge, our study is the first to identify the main clinical risk factors for DM in patients with SGC‐NLNM.

Nomograms are precise and useful clinical tools that can help clinicians predict the probability of an outcome event, such as survival time and LNM. A variety of nomograms have been built to predict the therapeutic benefits, postoperative survival rate, and LNM in patients with GC.^13^, ^14^, ^15^ Here, we constructed a nomogram to predict the risk of DM on the basis of the clinical characteristics of patients with SGC‐NLNM. To test the performance of our nomogram, ROC curves were generated in both the training and validation sets. According to previous studies, an AUC of the ROC curve >0.7 indicated that the built nomogram had a good accuracy and an acceptable discrimination.^16^, ^17^ In addition, calibration plots showed that the predicted DM probability was identical to the actual observation. Thus, the built nomogram incorporating clinicopathological characteristics was proved to have an appealing sensitivity and specificity in predicting the risk of DM.

Superficial gastric cancer seldom presents with DM, especially among patients without LNM. There are several metastatic patterns for GC, including lymphatic and hematogenous metastases. The latter pattern, where tumor cells invade blood vessels and directly metastasize to distant organs, might be the main routine for DM in SGC‐NLNM. As mentioned above, if without DM, most patients with SGC have a good prognosis after surgery or ET. Thus, it is urgent to determine the main risk factors of DM in patients with SGC‐NLNM to recognize the possibility of DM at the earliest. Routine imaging examinations such as magnetic resonance imaging and computed tomography detect obvious disseminated lesions, and positron emission tomography‐computed tomography is a more reliable method for DM screening in GC, especially in detecting micrometastasis.^18^, ^19^ However, the availability and the cost limit their usage. Therefore, it is meaningful to build a handy and economic nomogram that can help clinicians select high‐risk patients.

In our logistic regression model, we discovered that race, age, year of diagnosis, primary site, depth, size, and grade were independent risk factors for DM in patients with SGC‐NLNM. Most of the previous studies focused on independent variables associated with LNM in SGC and found that tumor depth, differentiation grade, size, and lymphatic invasion were closely associated with LNM.^20^, ^21^ Wang et al. reported that age was an independent LNM predictor in SGC, and LNM was relatively common in young patients.^22^ Similarly, our study found that patients ≤60 years old with SGC‐NLNM had a higher DM risk than those >60 years old. According to the Japanese Gastric Cancer Treatment Guidelines (fifth edition), a tumor size of 2 cm is an important factor when recommending ET for patients with SGC.^23^ Park et al. reported that when compared with tumor size ≤2 cm, the OR for LNM ranged from 1.04 to 2.36 in the group with a tumor size >2 cm in their multivariate analysis.^24^ In our multivariate logistic regression model, the groups with tumor size ≤3 and ≤5 cm had significantly higher DM risk when compared with the group with a tumor size ≤2 cm, suggesting that the tumor size was a powerful predictor for DM in patients with SGC‐NLNM. The built nomogram finally corroborated that the size of the tumor was one of the main risk factors in the prediction of DM.

Whether the Lauren type is an independent LNM risk factor is controversial.^25^, ^26^ Recently, a population‐based study revealed that diffuse‐ and intestinal‐type EGCs had a similar LNM risk and prognosis.^27^ In the present study, patients with diffuse‐type SGC‐NLNM had a comparable DM risk to those with intestinal‐type. For metastatic patients, our survival analysis found that diffuse‐ and intestinal‐type patients also had a comparable prognosis. Regarding the primary site, a previous study reported that the tumor location was not an LNM predictor for patients with SGC.^28^ In our study, the primary site was found to be a strong predictor of DM in patients with SGC‐NLNM. The primary sites of the cardia, gastric body, fundus, greater curve, and overlapping/NOS were associated with a higher risk of DM than the antrum/pylorus. It is well known that tumor depth is a strong predictor of LNM in SGC. The deeper the carcinoma infiltrates into the gastric wall, the higher the LNM risk.^29^ One possible reason is that a deeper mucosal infiltration means more lymphatic vessel involvement. In our present study, compared with the submucosa group, the proportion of DM was higher in the mucosa group; this might be explained by the leading metastatic pattern of lymph vessels in the submucosa layer, which indicates that a large proportion of patients with GC who have submucosa invasion might have presented with LNM and were excluded from our study.

Our survival analysis for patients with DM revealed that surgery (HR = 0.249; 95% CI, 0.125–0.495), chemotherapy (HR = 0.473; 95% CI, 0.353–0.633), and grade (HR = 1.374; 95% CI, 1.018–1.854) were independent prognostic factors associated with CSS. Palliative surgery and chemotherapy could significantly improve the prognosis of patients with metastatic SGC‐NLNM, while radiotherapy could not. Previous literature reported that the median survival time was only 13–16 months for metastatic GC.^30^ In our study, the median survival time for patients with metastatic SGC‐NLNM who underwent surgery was about 3 years. Therefore, for this special advanced GC, aggressive surgery and chemotherapy may be a better treatment approach than radiotherapy. Novel regimens such as targeted therapy and conversion therapy should be further explored to improve the survival rate for this rare entity in the future.

Our study has several limitations. First, because our study was a retrospective study and the inclusion of patients dated from 2004 to 2015, there remains the possibility of error related to miscoding and selection biases. Second, the population in our study was obtained from an American database; whether clinical characteristics of patients with SGC and DM risk are similar in other populations needs further multicenter investigation. Third, some variables, such as *Helicobacter pylori* infection status, demarcation line of tumor lesion, tumor markers, nutritional status, and Charlson Comorbidity Index may also be potential risk factors for DM in patients with SGC‐NLNM and need to be incorporated in our model. However, due to the unavailability of these variables in the SEER database, these variables could not be incorporated in our study. Their effects on DM in patients with SGC‐NLNM deserve further exploration. Incorporating other significant factors may further enhance the accuracy and effectiveness of the nomogram. Finally, confined by the limited cases and missing data in the SEER database, we were unable to carry out further stratified analysis of the metastatic site.

In conclusion, we constructed and validated a predictive clinical tool for DM in patients with SGC‐NLNM that can help clinicians select high‐risk patients with SGC‐NLNM who may harbor disseminated disease at initial diagnosis and guide appropriate metastatic screening plans. In our study, the tumor size and primary site were the largest contributors to the prediction of DM. Our study also found that for patients with DM, aggressive surgery and chemotherapy are better choices than radiotherapy.

## CONFLICTS OF INTEREST

None declared.

## AUTHOR CONTRIBUTION

JYC and LPW designed the study. ZZZ, SZ, and YFL collected and analyzed the data. JYC and ND wrote the paper. LHS and MX reviewed and revised the manuscript. The final manuscript was approved by all authors.

## ETHICAL STATEMENTS

The study used public data and followed the World Medical Association’s Declaration of Helsinki for Ethical Human Research.

## Supporting information

Table S1

## Data Availability

The data used and analyzed in our study can be downloaded from SEER (https://seer.cancer.gov/).
