# Supplementary material for: A clinical model to predict distant metastasis in patients with superficial gastric cancer with negative lymph node metastasis and a survival analysis for patients with metastasis
Source: Cancer Med. 2020 Dec 22;10(3):944–55. doi: 10.1002/cam4.3680 (PMC7897959; doi:10.1002/cam4.3680)
Supplement: Supplementary file 1 — Table S1 [file CAM4-10-944-s001.docx]

| **Supplementary table 1 Point assignment and predictive score in nomogram** | | | |
| --- | --- | --- | --- |
| Variable | Number | Score | Estimated metastasis probability |
| Age at diagnosis |  |  |  |
| ≤60 | 2 | 16 |  |
| >60 | 1 | 0 |  |
| Primary site |  |  |  |
| Antrumy/Pylorus | 1 | 6 |  |
| Body | 2 | 47 |  |
| Cardia | 3 | 46 |  |
| Fundus | 4 | 54 |  |
| Lesser curvature | 5 | 0 |  |
| Greater curvature | 6 | 43 |  |
| Overlapping/Nos | 7 | 51 |  |
| Grade |  |  |  |
| Well/Moderate | 1 | 0 |  |
| Poorly/Undifferentiated | 2 | 20 |  |
| Tumor size |  |  |  |
| ≤2cm | 1 | 0 |  |
| ≤3cm | 2 | 68 |  |
| ≤5cm | 3 | 80 |  |
| >5cm | 4 | 100 |  |
| Unknown/diffuse | 5 | 100 |  |
| Depth |  |  |  |
| T1a | 1 | 38 |  |
| T1b | 2 | 0 |  |
|  |  | Total predictive score |  |
|  |  | 75 | 0.01 |
|  |  | 139 | 0.05 |
|  |  | 168 | 0.10 |
|  |  | 200 | 0.20 |
|  |  | 221 | 0.30 |
|  |  | 238 | 0.40 |
|  |  | 254 | 0.50 |
